# Successful Management of First-Trimester Uterine Rupture and Placenta Previa: A Case Report

**DOI:** 10.7759/cureus.77857

**Published:** 2025-01-22

**Authors:** Yusuke Etori, Ryuhei Nagai, Yuta Shimomoto, Shinpei Yamamoto, Nagamasa Maeda

**Affiliations:** 1 Obstetrics and Gynecology, Kochi Medical School, Kochi, JPN

**Keywords:** definitive diagnosis, first-trimester, placenta accreta sequence, placenta previa, preventive intervention, symptomless, uterine rupture

## Abstract

A 36-year-old woman with five previous cesarean sections presented at 11 weeks of gestation with a 1 cm protruding lesion in the anterior vaginal fornix. Imaging, including MRI, revealed a myometrial defect covered by decidua, suggesting uterine rupture, alongside complete placenta previa. Despite high risks such as hemorrhage, further rupture, miscarriage, and preterm birth, the patient elected to continue her pregnancy under close surveillance. Weekly outpatient follow-up continued until 24 weeks, when transvaginal ultrasound examination showed uterine thinning, prompting hospital admission. A subsequent MRI confirmed the absence of fetal extrusion, and a multidisciplinary team readied for potential emergencies. At 29 weeks, worsening vaginal bleeding necessitated an urgent cesarean section. A 1,456-g male infant was delivered with Apgar scores of 2 at both one and five minutes; the placenta separated easily, and the uterine defect was sutured without incident. Notably, MRI findings ruled out placenta accreta by demonstrating a well-defined decidual layer. The patient’s asymptomatic presentation, despite a first-trimester rupture, raises the possibility of a chronic uterine defect existing before pregnancy. This rare case illustrates that even when faced with multiple prior cesarean sections, early uterine rupture, and placenta previa, meticulous imaging and coordinated multidisciplinary management can lead to a viable neonate and preserved maternal health. Further accumulation and review of such cases will help refine clinical guidelines for managing high-risk pregnancies.

## Introduction

Cesarean section is a crucial delivery method for ensuring the safety of both mother and fetus. However, a history of multiple cesarean sections increases the risk of serious complications such as uterine rupture and placenta accreta spectrum [1]. These complications often lead to severe hemorrhage and infection and may even result in a hysterectomy, posing a substantial threat to maternal and neonatal outcomes. In particular, patients with a history of four or more cesarean sections face a markedly heightened risk of severe maternal and fetal complications, necessitating careful risk assessment and strategic management [2]. However, it is challenging to diagnose uterine rupture and placenta accreta in early pregnancy definitively, and to avoid adverse maternal and fetal outcomes, there may be a tendency to overestimate the associated risks.

Here, we report a rare case in which a patient with five previous cesarean deliveries was diagnosed with uterine rupture at 11 weeks of gestation but opted to continue the pregnancy, ultimately delivering a live infant at 29 weeks. This report highlights the importance of diagnostic and therapeutic strategies in managing high-risk pregnancies.

## Case presentation

A 36-year-old woman (gravida 8, para 5) had previously undergone five cesarean sections, one induced abortion, and one spontaneous abortion. Neither her past medical history nor her family history revealed any notable findings. She conceived spontaneously and was followed at a local clinic.

**Figure 1:**
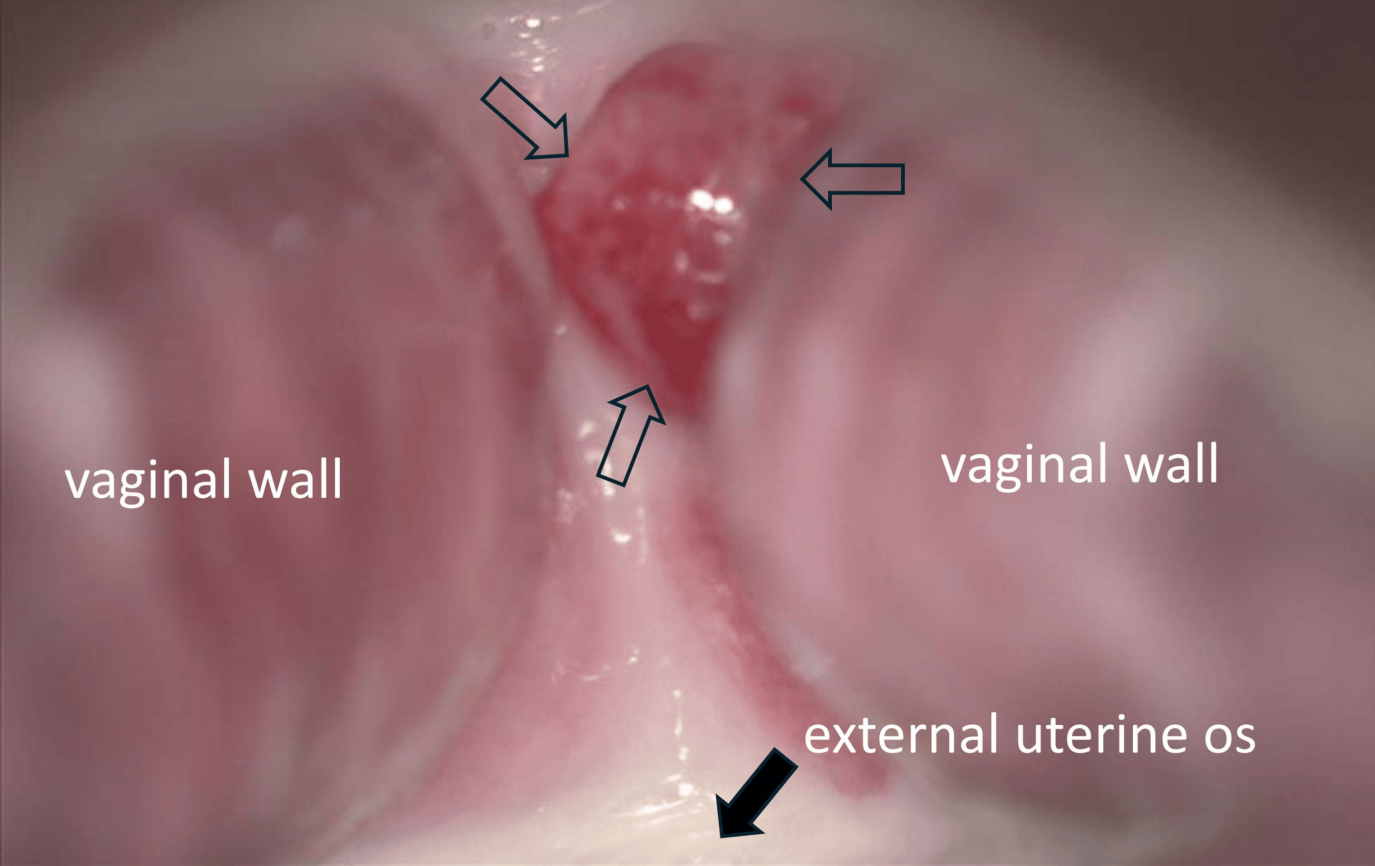
Tumorous lesion in the anterior vaginal fornix At 12 weeks of gestation, a red, soft, and easily bleeding raised lesion approximately 5 mm in size was observed in the anterior vaginal fornix (hollow arrows) located on the maternal abdominal side of the external cervical orifice (solid black arrow). At 14 weeks of gestation, a lesion of similar size was observed in the same location. However, by 16 weeks of gestation, the lesion was no longer detectable.

**Figure 2:**
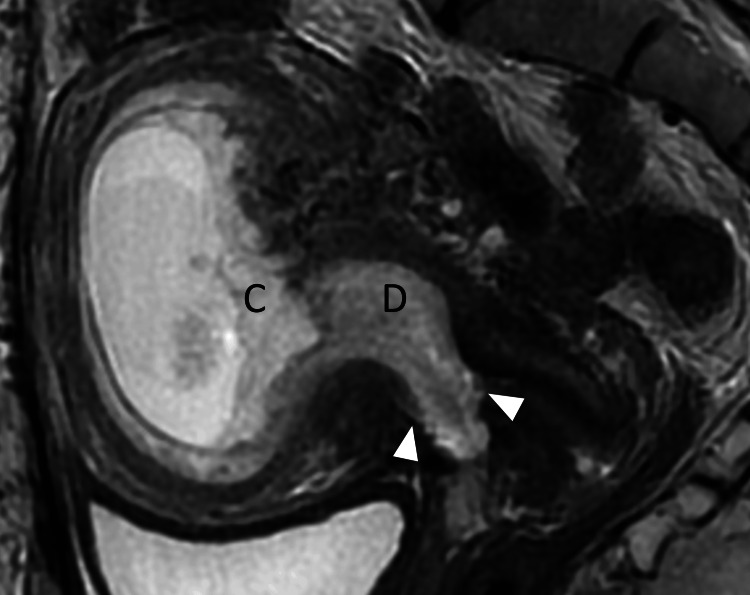
Sagittal T2WI MRI at 12 weeks gestation Absence of myometrium from the cervix to the anterior vaginal fornix (arrowhead). The myometrial defect site is observed to be infilled with decidual tissue (D), and chorionic villous tissue (C) is observed on the embryonic side of the defect, clearly separating the area from the decidual tissue. T2WI: T2 weighted image, MRI: magnetic resonance imaging

During a routine prenatal examination at 11 weeks of gestation, a red, protruding lesion measuring approximately 1 cm in diameter was observed on the vaginal mucosa of the anterior fornix by Cusco speculum examination (Figure 1). Grossly, the lesion resembled a decidual polyp and was clearly located on the vaginal mucosa anterior to the external os. Transvaginal ultrasound suggested a defect in the anterior cervical myometrium and detected vascularized tissue extending from the uterine cavity to the anterior vaginal fornix; however, a definitive diagnosis could not be made. Because of her history of five cesarean deliveries, uterine rupture was suspected, and an MRI was performed. The MRI confirmed a tear in the myometrium of the anterior vaginal fornix, consistent with suspected uterine rupture, and revealed that the defect was covered by decidua (Figure 2).

Furthermore, the placenta was found to be low-lying and covering the internal cervical os. Still, decidual tissue was present at both the internal os and the site of rupture, and no extrusion of chorionic or fetal components outside the uterus was observed. At 12 weeks of gestation, the patient was transferred to a tertiary perinatal care center for further evaluation and management planning.

Based on the clinical course and imaging findings, a diagnosis of uterine rupture and complete placenta previa was made. Should the pregnancy continue, the risks to the mother, including hemorrhage from the rupture site, possible extrusion of fetal tissues, and bleeding related to the placenta previa, were deemed extremely high. Similarly, the risks to the fetus, such as miscarriage or preterm birth and complications arising from uterine rupture, were also significant. Consequently, in addition to close monitoring aimed at continuing the pregnancy, termination of pregnancy was presented as an option. Although the patient’s family favored termination, the patient herself strongly wished to continue the pregnancy. Ultimately, after consultation with the perinatal care team, the decision was made to proceed with the pregnancy under strict surveillance.

**Figure 3:**
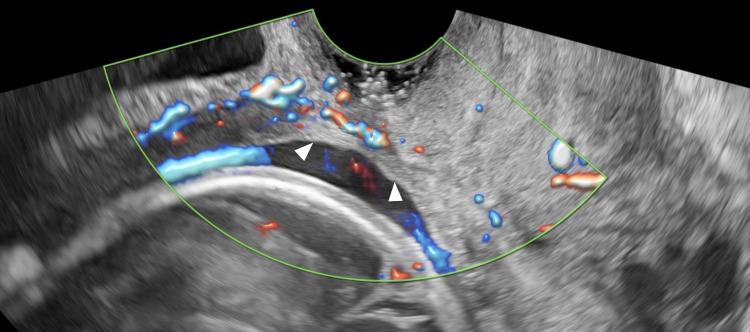
Sagittal transvaginal ultrasound color Doppler imaging at 24 weeks gestation The boundary between the decidual and chorionic tissue becomes thin and indistinct, and the area of myometrial defect is observed to widen (arrowhead).

**Figure 4:**
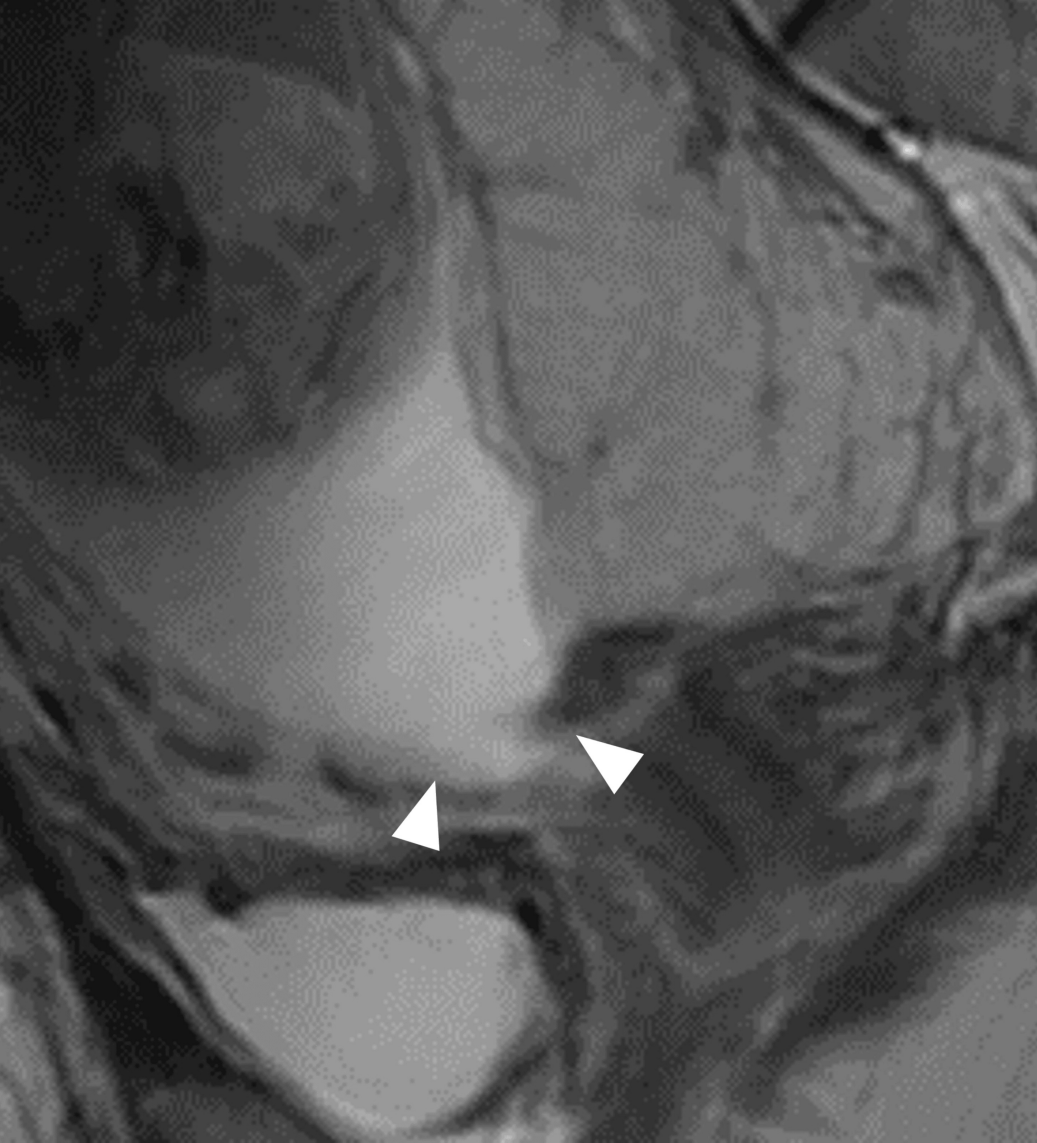
Sagittal T2WI MRI at 25 weeks gestation As in the ultrasound image, the decidual and chorionic tissues were thinning, and the boundaries were no longer discernible, but there was no evidence of fetal tissue bulging into the anterior vaginal fornix (arrowhead). T2WI: T2 weighted image, MRI: magnetic resonance imaging

After 14 weeks of gestation, weekly outpatient visits were conducted to monitor fetal growth and observe the rupture site via ultrasound, with a particular focus on myometrial thickness at the rupture site and any protrusion of fetal components. After 16 weeks of gestation, the decidual tissue protruding into the anterior vaginal fornix gradually regressed and eventually disappeared, rendering it no longer visible on examination of the anterior fornix. At 24 weeks, thinning of the uterine myometrium and a possible bulge of fetal tissue at the rupture site were suspected (Figure 3), prompting admission. A follow-up MRI at 25 weeks revealed generalized myometrial thinning but no extrusion of fetal components through the rupture site (Figure 4). The pregnancy appeared stable, and thus, a decision was made to prolong the gestation as much as possible.

In addition, a multidisciplinary conference involving obstetrics and gynecology, anesthesiology, pediatrics, radiology, urology, operating room nursing staff, and NICU nursing staff was held. During this conference, the timing of delivery and the required preparations, such as autologous blood donation, transfusion protocols, the potential use of interventional radiology for massive hemorrhage, and the timing of ureteral stent placement, were discussed and agreed upon in advance.

At 29 weeks and one day of gestation, the patient experienced vaginal bleeding suspected to be caused by placenta previa. Corticosteroids were administered in anticipation of possible preterm delivery, but the bleeding persisted and intensified, leading to an emergency cesarean section under general anesthesia at 29 weeks and four days. A male infant weighing 1,456 g was delivered, with Apgar scores of 2 at one and five minutes. Intraoperative findings revealed no evidence of placenta accreta, and placental separation was uncomplicated. The rupture site was covered with decidual tissue, and no fetal components had protruded. After placental removal, a defect in the uterine myometrium was palpated at the rupture site. On inspection using a Cusco speculum, a defect was noted in the anterior vaginal fornix mucosa with lochia drainage; thus, the area was sutured transvaginally using an absorbable suture. In this case, due to the high risk of complications in future pregnancies, such as uterine rupture and preterm birth, which could significantly affect both maternal and fetal outcomes, pregnancy was deemed medically inadvisable. Therefore, a preoperative plan was established to perform sterilization if surgical intervention became necessary, including the possibility of doing so during an emergency cesarean section. Furthermore, as the patient and her family did not wish to have any more children, a bilateral salpingectomy was ultimately performed. The total operative blood loss was 2,660 g, necessitating transfusion of four units of RBCs. However, maternal vital signs remained stable throughout the procedure. Approximately 10 minutes were required from the start of surgery to neonatal delivery, during which the neonate received an Apgar score of 2 at one minute and required intubation. Extubation, however, was planned for the following day, and no further significant complications were observed. The mother’s postoperative course was uneventful, and she was discharged following a recovery similar to that of a routine cesarean section. Notably, the uterus was preserved.

## Discussion

In this case, a patient with five previous cesarean deliveries experienced uterine rupture as early as 11 weeks of gestation, together with placenta previa, representing an extremely high-risk scenario. At first, the primary concern was whether to continue the pregnancy at all, given the considerable risks posed to both mother and fetus. Nonetheless, the patient was firmly committed to continuing the pregnancy. Following thorough explanations of the risks and benefits, a shared decision was reached to pursue a high-risk pregnancy management strategy. Placenta previa often leads to unpredictable, painless bleeding in the mid to late stages of pregnancy, and multiple prior cesarean deliveries markedly increase the likelihood of severe complications such as uterine rupture and massive hemorrhage. In this case, therefore, early and vigilant monitoring of the uterine defect was imperative, and from 24 weeks of gestation onward, the patient underwent regular ultrasound and MRI examinations to detect signs of fetal tissue protrusion or progression of the uterine defect.

**Figure 5:**
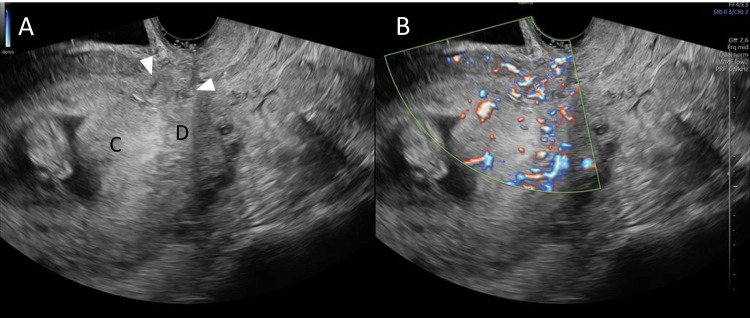
Sagittal transvaginal ultrasound B-mode imaging (A) and color Doppler imaging (B) at 12 weeks gestation (A) Similar to the MRI image, the lower myometrium is deficient, and the area is observed to have penetrating decidual tissue toward the anterior vaginal fornix (arrowhead). The boundary between the decidual tissue (D) and the chorionic villous tissue (C) can also be confirmed as in the MRI image, and by comparing the MRI image and the ultrasound image, a more detailed evaluation of the tissue characteristics can be performed. (B) When the same region is observed using color Doppler imaging, the decidual and chorionic villous tissue exhibit different blood flow patterns, making the boundary between them more distinct. MRI: magnetic resonance imaging

Of particular concern in patients with multiple prior cesarean sections is the possibility of placenta accreta (including increta or percreta) [3]. In this patient, MRI findings confirmed the presence of a distinct layer of decidua between the chorion and the rupture site, effectively ruling out placenta accreta, an especially noteworthy point that contrasts with previous reports. Moreover, correlating MRI images with ultrasound findings made it unnecessary to conduct multiple MRI scans, allowing ongoing monitoring via transvaginal ultrasound alone (Figure 5). Ultimately, at 29 weeks, the patient underwent an emergency cesarean section at a facility equipped with a NICU, enabling both mother and infant to achieve a stable outcome.

Another important consideration is that uterine rupture during the first trimester of pregnancy is exceptionally rare. In about 97% of reported cases, it presents with abdominal pain, whereas our patient displayed no such symptoms [4-6]. The operative report from the previous cesarean section noted significant adhesions between the bladder and uterus, making dissection difficult and wound closure challenging. Although speculative, the likely cause of both the anterior vaginal fornix defect and the myometrial thinning may be the inadequate repair of the already thinned myometrium during the closure of the previous cesarean incision, resulting in further thinning at that site. Moreover, it is possible that the synthetic absorbable sutures used for the repair inadvertently incorporated the anterior vaginal fornix mucosa, leading to fistula formation. It is thus speculated that a chronic uterine defect may have predated the pregnancy, persisting at the anterior vaginal fornix while remaining covered by decidua, which could account for the absence of acute symptoms. Although this remains a conjecture, this case demonstrates that even in situations where uterine rupture is detected early in pregnancy, diligent investigation and precise assessment of the etiology and pathology can result in continued pregnancy and a viable neonate.

## Conclusions

This case describes a highly unusual situation involving multiple previous cesarean deliveries, uterine rupture, and placenta previa. Yet, the patient successfully carried the pregnancy to 29 weeks through rigorous monitoring, a multidisciplinary team approach, and a combined imaging strategy employing MRI and transvaginal ultrasound. Early diagnosis of uterine rupture in the first trimester is extraordinarily difficult, and most such cases typically present with pain. The fact that our patient was largely asymptomatic yet managed under a robust high-risk protocol underscores the critical importance of flexible diagnostic and management strategies in complicated pregnancies. Further accumulation and examination of similar cases will be essential for establishing safer, more comprehensive guidelines for managing such extremely high-risk pregnancies.
